# *In silico* approach to predict candidate R proteins and to define their domain architecture

**DOI:** 10.1186/1756-0500-5-678

**Published:** 2012-12-08

**Authors:** Walter Sanseverino, Maria Raffaella Ercolano

**Affiliations:** 1 Department of Soil, Plant, Environmental and Animal Production Sciences, University of Naples “Federico II”, Via Università 100, Portici, 80055, Italy

**Keywords:** Disease resistance gene, Plant UniGene, Domain arrangements, Bioinformatics analyses

## Abstract

**Background:**

Plant resistance genes, which encode R-proteins, constitute one of the most important and widely investigated gene families. Thanks to the use of both genetic and molecular approaches, more than 100 R genes have been cloned so far. Analysis of resistance proteins and investigation of domain properties may afford insights into their role and function. Moreover, genomic experiments and availability of high-throughput sequence data are very useful for discovering new R genes and establish hypotheses about R-genes architecture.

**Result:**

We surveyed the PRGdb dataset to provide valuable information about hidden R-protein features. Through an *in silico* approach 4409 putative R-proteins belonging to 33 plant organisms were analysed for domain associations frequency. The proteins showed common domain associations as well as previously unknown classes. Interestingly, the number of proteins falling into each class was found inversely related to domain arrangement complexity. Out of 31 possible theoretical domain combinations, only 22 were found. Proteins retrieved were filtered to highlight, through the visualization of a Venn diagram, candidate classes able to exert resistance function. Detailed analyses performed on conserved profiles of those strong putative R proteins revealed interesting domain features. Finally, several atypical domain associations were identified.

**Conclusion:**

The effort made in this study allowed us to approach the R-domains arrangement issue from a different point of view, sorting through the vast diversity of R proteins. Overall, many protein features were revealed and interesting new domain associations were found. In addition, insights on domain associations meaning and R domains modelling were provided.

## Background

During their life plants are continuously under pathogen attack. Due to their nature, namely the lack of mobility, plants have developed molecular and chemical features to withstand biotic stresses. The plant immune system is based on receptors that recognise broadly conserved molecules associated to a wide range of pathogens. Resistance gene products (R proteins) are thought to recognise signal molecules produced by the pathogen and to respond by initiating rapid changes in host cell physiology and metabolism so as to directly inhibit pathogen growth.

To date, more than 100 R genes have been cloned (http://www.prgdb.org). Five typical protein structures were recognised as involved in the resistance process: the TIR-NBS-LRR (TNLs; e.g. *N* gene) [1], the CC-NBS-LRR (CNLs; e.g. *I2* gene) [2], the receptor-like kinase (RLKs; e.g. *FLS2*) [3], the receptor-like protein (RLPs; e.g. *CF4* gene) [4] and the kinase-like protein (e.g. *PTO* gene) [5]. The five R-protein types share common features: two of the five classes (RLK and RLP) contain a transmembrane domain (TM) that anchors them into the membrane, and four of them contain a leucine-rich repeat region (LRR) [6]. Classes TNL and CNL, lacking clear membrane anchor domains, operate mainly in the cytoplasm. Both contain a Nucleotide-Binding Site (NBS) and an LRR domain [7]. The TNL class has, additionally, a N-terminal domain with homology to the animal Toll-Interleukin Receptor (TIR). By contrast, the CNL class lacks the TIR domain and may include a C-terminal Coiled-Coil region (CC). Several RLK and RLP proteins confer resistance to biotic stresses. However, their function should be tested experimentally, because these proteins are involved also in other cellular mechanisms not related to resistance. RLKs consist of an intracellular serine kinase domain (KIN) and extracellular leucine-rich repeat region (eLRR) of 25-38 amino acids (AA) that confer a broad interaction surface, well suited to interact with multiple ligands [8]. The eLRR domain plays a recognising role, while the kinase triggers the downstream activation cascades [9]. RLKs can either function as homodimers [10] or require heteromeric interactions with other proteins to initiate a defence response [11,12]. Moreover, those genes can have multi-functionality activity [13]. Similar in function and structure, the RLP family consists of a serine/threonine receptor containing a leucine-rich region (KIN-LRR), a transmembrane region of ~25 AA, and a short cytoplasmic region, with no kinase domain [14]. The RLP extracellular leucine-rich repeat (eLRR) shows homology with the eLRR of the RLKs. Moreover, RLPs can be involved in other cellular mechanisms, like RLKs do [14]. Finally, proteins containing only a kinase (KIN) domain, like the tomato *PTO* gene [5] that confers resistance to *Pseudomonas syringae*, completes the panorama of R proteins. In addition to these well-studied five R-classes, many other resistance proteins (Oth-R), which exert their function in different ways, have been discovered. Sometimes they share conserved domains with the classified R proteins, but their functional mechanisms are usually so different that they cannot be simply classified [15,16]. In this class fall the *Hordeum vulgare MLO*[17] and the *Arabidopsis thaliana RPW8* genes [18], that confer resistance against the powdery mildew caused by *Blumeria graminis* and *Golovinomyces cichoracearum,* respectively. The study of this class of proteins may be of great interest to gain insights into the plant immune system overall [19].

For a long time R proteins were thought to recognize specific pathogen proteins using ill-defined mechanisms. Many models have been proposed to explain the way R proteins act, including the guard hypothesis, the zig-zag model and the switch model [20,21]. The most widely endorsed model connects various actors, assuming a collaborative role among PTI (PAMP-triggered immunity) proteins and resistance proteins [22]. By using domain architecture comparisons and domain property investigation, the role and function of R proteins and the ways for generating novelty may be better appraised. Genomic experiments, availability of sequenced genomes and high-throughput analysis can be very useful to discover new R-proteins and lay down new hypotheses concerning the domain reorganization process.

Recently, the plant resistance gene database (PRGdb, http://www.prgdb.org) has been developed. It is a specific resource collecting all functional R-genes and many putative sequences predicted by UNIGENE and NCBI nucleotide datasets. Prediction analysis using such data shows that plant genomes not only code for R proteins with known domain arrangements. but also for proteins with new resistance domain associations [23].

The aim of this paper is to revisit the information generated until now on R-proteins, analysing in depth the largest plant R UniGene dataset. We first analysed the frequency of domain associations to provide data on R-domain distribution and to discover new putative R-protein models. Then, after a manual curated data filtration, we highlighted features and levels of conservation among R-classes. Finally, we explored sequences similar to the neglected “other” R class (Oth-R) and with atypical R-domain associations (Aty-R).

## Results

### Analysis of R-domain associations in the UniGene PRG dataset

**Figure 1:**
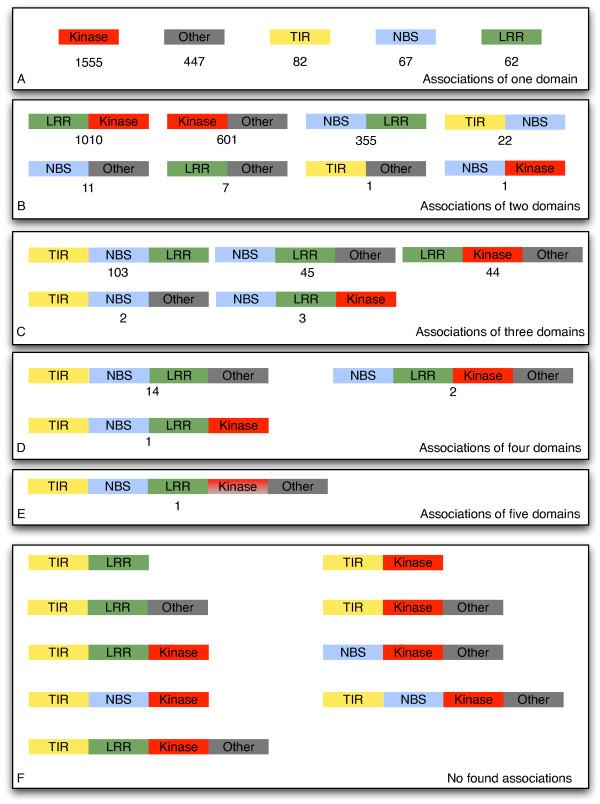
**R-domain associations found in the UniGene PRG dataset.** Predicted sequences are grouped according to the number of domains identified (**A-E**). Box **F** reports the associations not found in our dataset. LRR = Leucine-rich repeat; NBS = Nucleotide-binding site; TIR = Toll-interleukin like receptor; KIN = kinase.

The PRGdb full dataset was surveyed to identify UniGenes starting with proper initial codon (methionine). Of 10,463 translated proteins belonging to 33 organisms (UniGene eudicotyledons), 4409 were selected and classified according to their conserved domains. A contingency table (data not shown) allowed us to divide proteins into 22 subfamilies according with their domain composition. As shown in Figure 1, a considerable number of proteins are composed by a single domain (40%): 1555 proteins showed a kinase domain, 82 a TIR domain, 67 a NBS domain, 62 a LRR domain and 447 have domains classified as other (Oth-R). Associations comprising from two to five domains were also found. Few proteins showed a complex domain association comprising more than three domains. Most of the domain associations described here (42% of the total) were already reported in the literature. In particular, the already described classes are subdivided into kinases (KIN) (35.2%), transmembrane receptors (RLP or RLK) (22.9%), and cytoplasmic proteins (CNL and TNL) (9.2%). The protein domain arrangements not yet described ranged from plausible highly represented classes (e.g Kinase-OthR, 13%) to the non-ordinary class TIR-NBS-LRR-KIN-Oth-R (less than 1%). Looking at R-domain occurrence in the full dataset, the NBS domain was found in 13 classes, followed by the LRR domain in 12, the KIN domain in 9, and finally TIR domains in 8 classes. Preferential associations were observed between LRR-NBS and LRR-KIN domains that are present in 8 and 6 classes, respectively. Out of all possible combinations, nine were not found in our analysis. All of them, except one, contain a TIR domain (Figure 1F).

**Figure 2:**
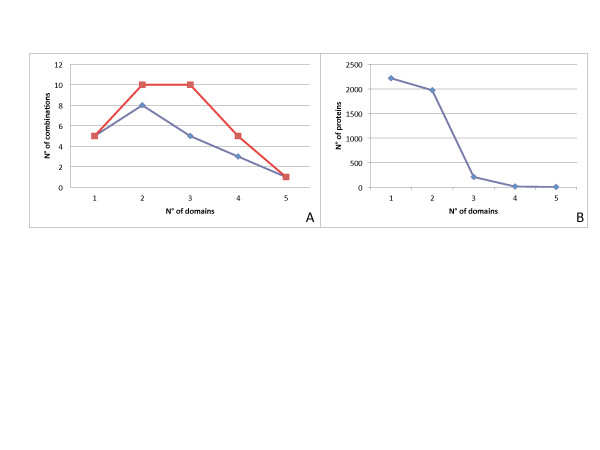
**A) Distribution of theoretical (red) and observed (blue) domain associations. B**) R proteins arranged in ascending order with corresponding observed frequencies.

To assess the biological relevance of the domain associations, we compared the theoretical and observed domain association distributions (Figure 2A). Overall, 31 hypothetical domain associations were displayed. Our theoretical model calculated by a binomial formula showed that proteins composed by three domains and two domains were the most numerous (33%), followed by one and four-domain associations (16%). Arrangements up to five domains should be rare. In our processed dataset only 22 out of 31 possible combinations were found. Five classes showed only one domain (20%), 8 classes two domains (37%), 8 classes three domains (25%), 3 classes four domains (11%), and 1 class five domains (2.7%). The distribution displayed by our sample, in comparisons with the theoretical distribution of proteins domain composition, indicates that R-domain combinations are not random in nature. Indeed, the number of proteins in each class is inversely related to domain arrangement complexity (Figure 2B).

### Data filtering

To perform a reliable R-proteins classification, data obtained from PRGdb has been filtered according to specific R-protein features. Our specific goal was to deeply annotate putative resistance genes and to find signatures that could support a resistance function. For the most important resistance class (NBS-LRR proteins) a coiled-coil prediction analysis, allowed us to divid into two sub classes: NBS-LRR (45 proteins) and CNL (310 proteins). Using the same procedure, proteins containing only a NBS domain have been divided into CC-NBS (30 proteins) and NBS (37 protein). Instead a transmembrane motif prediction allowed us to divide transmembrane receptors into two classes: RLK (transmembrane motif in the middle of protein) and RLP (transmembrane protein at the C terminal of protein). In order to extract receptors putatively involved in resistance processes RLP proteins have been filtered according to the Fritz-Laylin method. Proteins containing only a LRR domain (LRR-Oth-R, LRR-KIN-Oth-R) have been deleted and kinases have been filtered to select only PTO-like proteins. A total of 107 RLP proteins close related to previous cloned R proteins and 17 proteins containing the PTO-like domain were identified (PTHR24420:SF785 or PTHR23258:SF418, Panther database). In addition, proteins containing an Oth-R domain have been filtered to select only MLO-like and RPW8-like proteins. Seventy-five proteins containing the MLO-like domain (PF03094, Pfam database) and 7 proteins possessing the RPW8 domain (PF05659, Pfam database) were selected. RPW8-like proteins have been divided in two sub-classes: proteins containing only the RPW8 domain and proteins containing a RPW8 domain associated with an NBS-LRR profile (see “Atypical association paragraph”). The 75 MLO-like proteins have been phylogenetically analysed to extract 11 MLO-like proteins that cluster in the same clade of the *Arabidopsis* and tomato MLOs functional genes [17]. Finally, to confirm the cellular localization of R proteins, a transmembrane region prediction was performed on all the selected candidates. Through a combined *in silico* approach, using Phobius, TMHMM v2.0 and Interproscan tools, each protein was scanned for the presence of transmembrane domains (TM). Interestingly, transmembrane signatures have been found in cytoplasmic classes (Table 1).

**Table 1:** Proteins containing putative transmembrane motifs

**R-domain associations**	**n. TM**	**% TM**	**1 TM**	**2 TM**	**More TM**
CNL	20	6.4%	17	3	0
RLP	107	100%	63	41	3
TNL	17	16.5%	12	3	2
TIR	9	10.9%	6	2	1
NBS-LRR	1	2.2%	1	0	0
NBS	3	8.1%	8	0	0
TIR-NBS	2	9%	0	2	0
NBS-OthR	1	8.3%	1	0	0
PTO-like	9	56.2%	1	8	0
MLO-like	11	100%	0	4	7
TNL-OthR	1	7.1%	0	1	0

For each class we report the number of sequences with a TM signature (n.), the percentage of sequences with a TM motif on the total (%) and the number of sequences with one, two or more TM domains.

**Figure 3:**
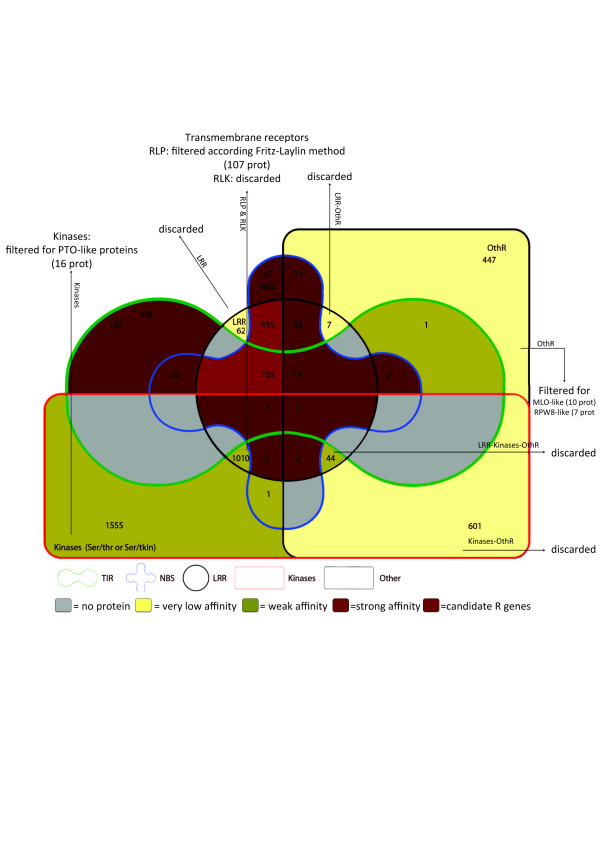
**Edwards Venn diagram in which proteins are grouped according to their interpro-scan profile.** The edge colors represent the five domains [LRR, Kinase, NBS, Other (OthR) and TIR] reported in legend. The group’s intersections showed all possible domain combinations. The colour filling intensity indicated the affinity of a determinate group to exert a resistance function. Stronger colours showed a high probability to exert only a resistance function; Purple red has been used for the CNL and TNL families. The families that could exert a resistance function, but have been not yet described in the literature, were brown coloured. Green and yellow colours have been used for families that need further analysis to validate their putative resistance function. Grey areas showed classes not found in our dataset. Numbers indicated proteins belonging to a specific group. Arrows were used to highlight the analyses performed for a given group in order to select further R-protein candidates.

To better appreciate the overall filtering process, an Edwards Venn diagram has been drawn where proteins are divided according to our annotation. Using information about the predicted function of each domain, different groups have been obtained (Figure 3). Out of 4409 proteins, 817 strong R-candidates have been selected. Proteins coloured with darker colours showed a high probability to exert merely a resistance function. Purple red has been used for the CNL and TNL families that are the best candidates to exert a resistance function. The families that could exert a resistance function, but have not been described yet in the literature as functional classes, were brown coloured. Green and yellow colours have been used for families that need further analysis to validate their putative resistance function. Among them, 76 proteins with new domains or with a new domain association were found. Grey areas showed classes that were not found in our dataset.

### Resistance families’ comparison

Classes with more than 10 sequences were analysed for their level of similarity in order to reveal domain features and the level of conservation within members belonging to the same class (Table 2). The length of proteins ranged from 1146 AA of TNL protein to 226 AA of proteins with only a TIR domain. The number of members belonging to each class ranged from 310 (CNL) to 10 (MLO-like proteins). Class identity ranged from 14.1% to 55.5%. Identical amino acid sites were not found in RLP class. Comparisons of multiple alignments of random samples, supported by an ANOVA test, revealed that identity is unaffected by number and length of aligned sequences.

**Table 2:** Multiple alignment comparison of 10 R protein groups composed by typical resistance domains

**Class Name**	**n. of sequences**	**Average sequence length**	**Alignment length**	**Identity**	**Identical sites**
CNL	310	1014	5050	16.3%	0.3%
RLP	107	651	1734	17.1%	0.0%
TNL	103	1146	3717	25.6%	0.1%
TIR	82	226	801	29.3%	0.5%
NBS-LRR	45	831	2750	15.4%	0.4%
NBS	37	361	1070	14.5%	0.1%
CC-NBS	30	395	804	16.4%	0.1%
TIR-NBS	22	502	833	31.8%	1.0%
PTO-like	16	561	921	35.7%	6.0%
MLO-like	10	427	622	55.5%	14.1%

Groups were ordered according to the number of retrieved sequences. Average sequence lengths, percentage of identity and percentage of identical sites are reported.

**Figure 4:**
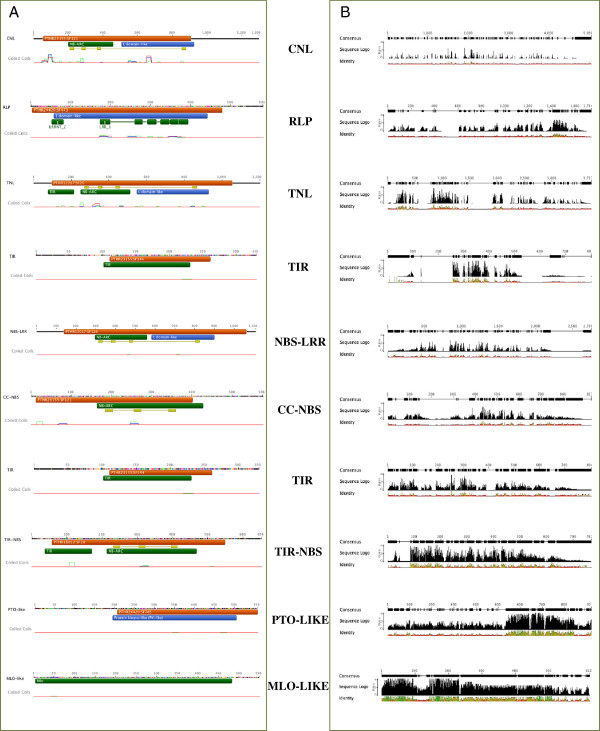
A) Interproscan results of consensus sequences of each analysed class B) Pattern of conservation along consensus sequence among members of each class.

InterProScan protein signature check allowed us to underline conserved domains along the proteins (Figure 4). The pattern of conservation within each group provided evidence that the most conserved classes are the single protein PTO-like and MLO-like, followed by those in which a TIR domain is present: TNL, TIR, TIR-NBS. By contrast, the less conserved are those in which the LRR domain is present. In general, the LRR profile showed a high variability in typology and number of repetitions among proteins. Moreover, in RLPs the LRR is positioned at the N-terminal, while in CNL and TNL at the C-terminal. RLP proteins showed a consensus starting motif MALS(L) _n_ and a high level of conservation at the kinase level. The NBS domain is more conserved in TNLs than in CNLs. TIR is the most conserved R domain, with the peak of conservation in N-terminal that reveal a common starting motif MA(S)_n_ and a core conserved domain region. Also for this domain differences among single classes can be evidenced. The TIR domain is more conserved in the TIR-NBS class and in proteins not characterized by other signatures. Moreover, proteins composed by only one TIR domain showed differences in the last part of the profile (Figure 4).

### R domain atypical associations (Aty-R)

Interproscan annotation of putative resistance proteins evidenced the presence of proteins containing unreported domains associated with a R domain. Table 3 shows the most interesting associations found in our dataset. In *Arabidopsis* the RPW8 domain, known to be involved in the resistance against powdery mildew, was found associated with NBS and LRR domains. Moreover, some of the “Aty-R” domains identified in this work overlap R domains, indicating that they could play a similar role. An example is the TIRAP domain, a Toll/Interleukin-1 Receptor domain containing an adaptor protein, present in *Arabidopsis thaliana*, *Vitis vinifera* and *Gossypium hirsutum,* which showed a very similar sequence to the TIR domain. It has not been found yet in the resistance gene families. Interestingly, some associations were found only in certain species, whereas others were ubiquitous. The association between NBS and “Aty-R” only occurred in *Oryza sativa*. Retrotransposon elements in association with the R-gene domain were also found. In particular, seven proteins of *Oryza sativa*, four of *Populus trichocarpa* and one of *Arabidopsis thaliana* were evidenced (Table 4).

**Table 3:** Atypical R-domain combinations found in the UniGene PRGdb dataset

**Unigene ID**	**Resistance Class**	**Aty-R domain(s)**	**Species**
At.43365	NBS-LRR-AtyR	Mob1	*Arabidopsis thaliana*
Os.24417	NBS-LRR-AtyR	Zinc finger, ZZ-type	*Oryza sativa*
Os.25168	NBS-AtyR	GTPase Containing Family	*Oryza sativa*
Os.25222	NBS-AtyR	Phosphatase 2C	*Oryza sativa*
Os.25268	NBS-AtyR	WRKY transcription factor; Gag-Pol-related Retrotransposon	*Oryza sativa*
Os.27097	NBS-AtyR	WRKY transcription factor	*Oryza sativa*
Os.78619	NBS-AtyR	Zinc finger, CCHC-type	*Oryza sativa*
Os.79969	NBS-AtyR	DUF1979	*Oryza sativa*
Os.93921	NBS-AtyR	Cecropin; Origin replication binding protein	*Oryza sativa*
Pth.15498	NBS-AtyR	Gag-Pol-related Retrotransposon	*Populus trichocarpa*
Pth.15636	NBS-AtyR	Zinc finger, BED-type	*Populus trichocarpa*
At.3076	TIR-LRR-NBS-KIN-Serthr-AtyR	Phenylalanine Hydroxylase (PAH); WRKY transcription factor	*Arabidopsis thaliana*
At.38115	TIR-AtyR	DUF541	*Arabidopsis thaliana*
At.51652	TIR-AtyR	Toll-IL-1 receptor domain-containing adapter protein (TIRAP)	*Arabidopsis thaliana*
Ghi.9199	TIR-AtyR	Toll-IL-1 receptor domain-containing adapter protein (TIRAP)	*Gossypium hirsutum*
Gma.3221	TIR-AtyR	Helix-loop-helix structural domain **(**EF-HAND 2)	*Glycine max*
Vvi.2456	TIR-AtyR	Toll-IL-1 receptor domain-containing adapter protein (TIRAP)	*Vitis vinifera*
At.46853	TNL-AtyR	Pleckstrin homology domain (PH); Regulator of chromosome condensation	*Arabidopsis thaliana*
Pth.16040	TNL-AtyR	CALCINEURIN B	*Populus trichocarpa*
Pth.16041	TNL-AtyR	DNA-Directed RNA Polymerase II	*Populus trichocarpa*
Pth.16058	TNL-AtyR	Gag-Pol-related Retrotransposon	*Populus trichocarpa*
Han.34	TIR-NBS-AtyR	Steroid Binding Protein	*Helianthus annuus*
Psi.4721	TIR-NBS-AtyR	Toll-IL-1 receptor domain-containing adapter protein (TIRAP)	*Picea sitchensis*

Sequences are divided according to the R domain and sorted by species.

**Table 4:** Candidate R genes showing a transposon insertion

**Gene Name**	**Species**	**Class or domain**
*Os.25268*	*Oryza sativa*	CNL-WRKY
*Os.53813*	*Oryza sativa*	CNL
*Os.78767*	*Oryza sativa*	Ser/thr
*Os.79795*	*Oryza sativa*	CNL
*Os.79928*	*Oryza sativa*	RLP
*Os.79975*	*Oryza sativa*	LRR
*Os.83660*	*Oryza sativa*	RLP
*Pth.15498*	*Populus trichocarpa*	NBS
*Pth.16071*	*Populus trichocarpa*	TNL-TIR
*Pth.16077*	*Populus trichocarpa*	TNL-TIR
*Pth.8196*	*Populus trichocarpa*	TNL

Sequences are divided according to species and resistance classes. Gene names correspond to UniGene Id.

## Discussion

The analysis performed in this paper sheds light on the complex panorama of resistance proteins, highlighting the “underground” information of this family. Although R-proteins are an important and useful family in plant species, some of their characteristics have not been elucidated yet [6,24]. With the advent of the genomic era, the classification of R-proteins into five families is now at odds with the latest discoveries in this field. From our data a possible new scenario emerges, where a broader repertoire of proteins might be involved in the resistance process. In the PRG UniGene dataset, using semi-automated prediction analysis, we detected proteins that were similar to functional R proteins. By choosing only UniGene sequences (set of tailed transcript sequences from the same locus), we avoided selecting pseudo-genes or predicted sequences derived from annotation errors. To ensure that our sampling was sufficiently accurate, the analysis was made more rigorous, selecting a subset of 4409 UniGene homologues to R-proteins and starting with a methionine. The use of a specific R-proteins prediction tool allowed us to place a large number of sequences in known R classes. However, numerous sequences similar to R proteins but with unknown domain arrangements were identified, including new associations among known R domains, proteins with a R domain repetition and sequences containing just one R domain.

Since protein domains are major evolutionary units, the identification of domain loss, transfer, duplication and combination with other domains to form new proteins is important [25]. The distribution of domain associations could be affected by natural pressure that somehow acts to select the most favourable associations to achieve a given task. Domains are considered to be the basic unit of proteins, and reorganizing these blocks may lead to significant changes in the physical structure as well as the biochemical activity of the corresponding proteins [26]. In our data, out of 31 theoretical combinations, only 22 associations were found. Interestingly, the observed data lack TIR associations. Domain shuffling was found to have an important role in the evolution of innate immune systems in both vertebrates and invertebrates [27]. In our study, a high number of proteins with one or two domains were found. R proteins could exert their function associated in a multi-protein complex or alone [28]. Proteins with multi-domains should be able to offer all specific needs for resistance (recognition, signal transduction and energy sourcing), while single domain proteins could change conformation more easily to be able to work in a protein complex. It may be more advantageous for living organisms to produce a higher number of proteins that allow flexible associations. Indeed, recent data suggest that the R domains need to be separated to exert their function [29-31]. Moreover, in our dataset, TIR-LRR, TIR-Ser/thr and more complex derived combinations were not found. These findings suggest that non-detected combinations may not be advantageous. Each domain has a specific function: LRR is involved in recognition and intramolecular interactions [21], kinase in signal transduction [32], NBS in ATP binding [33] and TIR in signalling and molecular interaction [34,35]. Described R domains seem to be essential to initiate a defence response in different patho-systems, but they can be associated in different ways. The associations evidenced in this work offer the opportunity to explore the full panorama of R proteins and understand the rationale of domain association.

In order to further characterize a subset of strong putative R-proteins, a filtering process was conducted. The most difficult part of the process was to find an efficient way to select good candidates to exert resistance function, loosing as few sequences as possible. The construction of a Venn diagram for visualizing the probability of proteins to exert resistance function based on the presence of “strong putative domain” and “weak putative domain” was very useful. Following this approach we were able to highlight putative disease resistance proteins. More detailed analyses were conducted only on proteins showing at least one domain homologous to domains identified in proteins with undisputed resistance function as reported elsewhere [8,24].

Proteins localization could affect domain function, activity, protein structure and affinity for other proteins. Hence, to outline the localization and conformations of putative R-proteins, we performed a transmembrane prediction. Some typical cytosolic classes like the CNL and TNL evidenced transmembrane domains. In order to verify evinced attributes more detailed studies should be performed.

Focusing on single protein class conservation pattern we evidenced some peculiar features. Indeed, little alterations of motifs could have a considerable effect on the functional specificities of the corresponding domains. The LRR domain was the most variable R domain in terms of number of leucine repetitions, length and conservation. The LRR domain is a common motif in more than 2000 proteins. At least four different families, LRR_1, LRR_2, LRR_3, FNIP [36-39], have been found. Differences in number and motifs composition among plant R proteins have been already reported [6]. Looking at R proteins, it is important to underline that the localization of this domain in the CNL/TNL classes and the RLP class is different. In the first case LRR repetitions are positioned at the C-terminal of the proteins, while in the second one at the N-terminal. These data suggest the occurrence of a different evolutionary process for the CNL/TNL and RLP classes, even if they share a common domain. NBS and TIR showed a high percentage of conservation to preserve their function as well as the RLP kinase domains. The NBS domain, associated with the TIR domain (TNL and TIR-NBS classes), is more conserved than the NBS found alone and the NBS present in the CNL proteins. In an *Arabidopsis* survey, the NBS domain of TNL and CNL are clearly distinguished in different phylogenetic branches [7]. Moreover, the NBS domain of TNL is reported to contain an additional loop [40]. Interestingly, the conservation profile of proteins characterized only by the TIR domain showed some specific peculiarities at the C-terminal part [16].

Finally, many sequences evidenced associations of R domains with domains involved in other processes or domains with unknown functions. Novel identified proteins were collected in a catalogue termed Aty-R (atypical resistance proteins). Several sequences often have a R domain with an additional motif. Interestingly, proteins with a WRKY motif (a motif found in zinc-finger, transcription factor and present also in the *RRS1* R-protein, [41]) were found. Aty-R domain associations could have occurred to improve specificity of the protein without changing its structure (TIRAP domain similar with TIR, STRUMBLING receptor similar to Ser/thr) or could have become established to enhance protein expression and stability through domains like WRKY, Zinc finger and EF-Hand [41].

The discoveries of domain associations and the presence of R-domains integrated in transposon elements enhance the possible organizations of R genes, adding new information on the feature of this family. Interestingly, a peculiarity for each species was found, namely the presence of transposon elements in the *Oryza* and *Populus* dataset. In *Oryza sativa* a transposon insertion in genes involved in the resistance process has already been found [42]. Besides transposon insertions, an association composed by TNL-TIR (repetition of TIR domain in the final part of the proteins) was found in *Populus trichocarpa*. A TIR-TIR interaction between the *N* and *N*^*tr*^ genes was revealed in *Nicotiana,* suggesting that two TIR domain interactions could increase resistance ability [43]. Overall, many protein features were revealed and interesting new domain associations were found.

The analysis performed in this study paves the way to understand how plant resistance domain associations are originated. Insights on R domains modelling were also provided. The panorama of R candidate proteins emerging from this analysis makes the current R-protein classification too restrictive. In addition, the recent increasing number of functional R-proteins found, difficult to classify, is a clear indication that a revision is needed [8,37].

## Conclusions

The purposes of our study were to investigate the domain architecture of translated expressed sequences similar to R-proteins and to develop approaches to identify candidates for functional studies. We believe that this work is the starting point to explore the panorama of resistance proteins within a different perspective. From our data it emerges that there are several aspects that merit an in-depth study. Tools should be developed to better discriminate general plant receptors from receptors involved in resistance process, to visualize new domain arrangements, to analyse possible 3D domain interactions and to provide models of action. Within the complex R-proteins scenario, our data pose new questions concerning the absence of some domain combinations, the role of sequences containing single domains, the possible involvement of new classes in the resistance process, the role of tandem R domains and the role of transposons in the functionality and expression of R genes. All analysed proteins and all produced datasets were available in a special section of the PRGdb (http://www.prgdb.org), with downloadable data, in-depth studies and advanced search method to extract specific proteins of interest.

## Methods

### Data selection

Overall, we inspected 10463 UniGene sequences, similar to proteins that exert resistance function, annotated through a specific R-protein prediction pipeline. The dataset was selected by the full NCBI UniGene plant dataset of 600,000 sequences translated by Estscan v.3.0.2 and analysed by the DRAGO pipeline [23]. From PRGdb with *ad hoc* queries, 4409 proteins starting with a methionine were extrapolated from the entire set and divided, according to their domains, into different classes.

Through an exhaustive data filtering system, a total of 817 proteins have been selected as strong putative resistance proteins. Ubiquitous proteins involved also in other cellular processes (like LRR, RLK, LRR-OthR, LRR-KIN-OthR and KIN-OthR classes) have been excluded from our dataset and stored in separate files. OthR, RLP and kinases classes have been filtered with specific phylogenetic and interproscan analyses.

### Protein analysis

Sequences were analysed with InterProScan 4.8 stand-alone version with the last update (spring 2011) of all 13 integrated databases (PROSITE, PRINTS, Pfam, ProDom, SMART, TIGRFAMs, PIR super family, SUPERFAMILY Gene3D, PANTHER and HAMAP) [44]. The output of each sequence was semi-manually checked for conserved domains and 4409 proteins were divided according to their conserved features. To classify putative R proteins in accordance with domain occurrence, a contingency table was obtained using R statistical software [45]. The total set of proteins was examined for the presence of transmembrane domains using Phobius [46] and TMHMM Geneious tools [47] while the coiled-coil prediction was performed by the coiled-coil tool of Geneious [47]. Proteins with new domain associations or containing domains involved in the resistance mechanism but not specific for it were manually inspected for discovering new R protein features. Data were recorded and used for further investigations.

### Domain associations

All possible R-domain combinations were calculated using the following binomial formula:

$$\sum_{k=1}^{5}\left(\begin{array}{l} n\\ k \end{array}\right)=\sum_{k=1}^{5}\frac{n!}{k!•\left(n-k\right)!}$$

in which *n* is the number of different domains and *k* the number of domains that can be found in a single protein. The distribution of theoretical R-domain associations was used to perform a comparison with our dataset. In this study the number of domain (*n*) is 5 and the number of domains that can be found in a single protein (*k)* is between 1 and 5.

### RLPs filtering

RLP reference resistance proteins (downloaded from PRG selecting “RLP class reference set”), RLPs predicted from our previous analysis and RLP not involved in resistance process were aligned with Muscle v3.6 [48] using a maximum number of iteration of 32. The transmembrane C3-F domains [14] was extracted and the alignment refined. This aligned region was used for a phylogenetic analysis with PHYML v3.0, using the JTT substitution model, transition/transversion model estimated, proportion of invariable site estimated, gamma distribution estimate and number of substitution for categories equal to 4. A tree/length/branch optimization has been obtained and accuracy has been calculated with aLRT statistics method [49]. This approach allowed us to separate RLPs homologues to reference resistance RLPs from others.

### MLO filtering

A phylogenetic analysis was performed on the predicted MLO-like proteins to select MLO- proteins that can confer resistance. The MLO reference resistance gene (http://prgdb.crg.eu/gene.php?id=35723&type=ref) [17] and three *Arabidosis* MLO-like proteins phylogenetically closed to it [50] were downloaded. The 75 MLO-like proteins predicted with our pipeline were aligned with references genes [50] for performing a phylogenetic analysis, following the procedure described in previous paragraph. Proteins belonging to same clade of MLO reference resistance gene have been selected.

### Pairwise identity and ANOVA test

Associations of known R domains consisting of more than 10 sequences were grouped and analysed for identity. The alignments were performed with MUSCLE v.3.6 with a maximum of 16 iterations. A total of ten groups of aligned proteins were obtained. Alignments were manually checked and unaligned regions were discarded. ANOVA analysis at 0.05 and 0.01 level of significance was performed on identity results obtained on 10 random batches of 10 sequences collected within each class and compared with results obtained using the total number of proteins belonging to each class. The conservation profile of each group was obtained and examined.

## Competing interests

The authors have declared that no competing interests exist.

## Authors’ contributions

Conceived and designed the experiments: MRE and WS; Performed data analysis: WS; Analysed results: MRE; Wrote the paper: MRE and WS. Both authors read and approved the final manuscript.

## References

[B1] Erickson FL Holzberg S Calderon-Urrea A Handley V Axtell M Corr C Baker B The helicase domain of the TMV replicase proteins induces the N-mediated defence response in tobacco Plant J 1999 18 1 67 75 10.1046/j.1365-313X.1999.00426.x 10341444

[B2] Ori N Eshed Y Paran I Presting G Aviv D Tanksley S Zamir D Fluhr R The I2C family from the wilt disease resistance locus I2 belongs to the nucleotide binding, leucine-rich repeat superfamily of plant resistance genes Plant Cell 1997 9 4 521 532 9144960 10.1105/tpc.9.4.521 PMC156936

[B3] Gomez-Gomez L Boller T FLS2: an LRR receptor-like kinase involved in the perception of the bacterial elicitor flagellin in Arabidopsis Mol Cell 2000 5 6 1003 1011 10.1016/S1097-2765(00)80265-8 10911994

[B4] Thomas C Jones D Parniske M Harrison K Balint-Kurti P Hatzixanthis K Jones J Characterization of the tomato Cf-4 gene for resistance to Cladosporium fulvum identifies sequences that determine recognitional specificity in Cf-4 and Cf-9 The Plant Cell Online 1997 9 12 2209 10.1105/tpc.9.12.2209 PMC157069 9437864

[B5] Martin G Brommonschenkel S Chunwongse J Frary A Ganal M Spivey R Wu T Earle E Tanksley S Map-based cloning of a protein kinase gene conferring disease resistance in tomato Science 1993 262 5138 1432 1436 10.1126/science.7902614 7902614

[B6] van Ooijen G van den Burg HA Cornelissen BJC Takken FLW Structure and function of resistance proteins in solanaceous plants Annu Rev Phytopathol 2007 45 43 72 10.1146/annurev.phyto.45.062806.094430 17367271

[B7] Meyers B Kozik A Griego A Kuang H Michelmore R Genome-wide analysis of NBS-LRR-encoding genes in Arabidopsis Plant Cell 2003 15 4 809 10.1105/tpc.009308 12671079 PMC152331

[B8] Shiu SH Bleecker AB Plant receptor-like kinase gene family: diversity, function, and signaling Sci STKE 2001 2001 113 re22 11752632 10.1126/stke.2001.113.re22

[B9] Bent A Plant disease resistance genes: function meets structure Plant Cell 1996 8 10 1757 1771 12239361 10.1105/tpc.8.10.1757 PMC161313

[B10] Morillo S Tax F Functional analysis of receptor-like kinases in monocots and dicots Curr Opin Plant Biol 2006 9 5 460 469 10.1016/j.pbi.2006.07.009 16877029

[B11] Weber ANR Moncrieffe MC Gangloff M Imler J-L Gay NJ Ligand-receptor and receptor-receptor interactions act in concert to activate signaling in the Drosophila toll pathway J Biol Chem 2005 280 24 22793 22799 10.1074/jbc.M502074200 15795223

[B12] Karlova R Boeren S Russinova E Aker J Vervoort J de Vries S The Arabidopsis SOMATIC EMBRYOGENESIS RECEPTOR-LIKE KINASE1 protein complex includes BRASSINOSTEROID-INSENSITIVE1 Plant Cell 2006 18 3 626 638 10.1105/tpc.105.039412 16473966 PMC1383638

[B13] Masle J Gilmore S Farquhar G The ERECTA gene regulates plant transpiration efficiency in Arabidopsis Nature 2005 436 7052 866 870 10.1038/nature03835 16007076

[B14] Fritz-Laylin L Krishnamurthy N Tor M Sjolander K Jones J Phylogenomic analysis of the receptor-like proteins of rice and Arabidopsis Plant Physiol 2005 138 2 611 623 10.1104/pp.104.054452 15955925 PMC1150382

[B15] Brandwagt BF Mesbah LA Takken FL Laurent PL Kneppers TJ Hille J Nijkamp HJ A longevity assurance gene homolog of tomato mediates resistance to Alternaria alternata f. sp. lycopersici toxins and fumonisin B1 Proc Natl Acad Sci USA 2000 97 9 4961 4966 10.1073/pnas.97.9.4961 10781105 PMC18340

[B16] Romer P Hahn S Jordan T Strauss T Bonas U Lahaye T Plant pathogen recognition mediated by promoter activation of the pepper Bs3 resistance gene Science 2007 318 5850 645 648 10.1126/science.1144958 17962564

[B17] Buschges R Hollricher K Panstruga R Simons G Wolter M Frijters A van Daelen R van der Lee T Diergaarde P Groenendijk J The barley Mlo gene: a novel control element of plant pathogen resistance Cell 1997 88 5 695 705 10.1016/S0092-8674(00)81912-1 9054509

[B18] Xiao S Ellwood S Calis O Patrick E Li T Coleman M Turner JG Broad-spectrum mildew resistance in Arabidopsis thaliana mediated by RPW8 Science 2001 291 5501 118 120 10.1126/science.291.5501.118 11141561

[B19] Jones J Dangl J The plant immune system Nature 2006 444 7117 323 329 10.1038/nature05286 17108957

[B20] Dangl JL Jones JD Plant pathogens and integrated defence responses to infection Nature 2001 411 6839 826 833 10.1038/35081161 11459065

[B21] Takken FLW Tameling WIL To nibble at plant resistance proteins Science 2009 324 5928 744 746 10.1126/science.1171666 19423813

[B22] Boller T He SY Innate immunity in plants: an arms race between pattern recognition receptors in plants and effectors in microbial pathogens Science 2009 324 5928 742 744 10.1126/science.1171647 19423812 PMC2729760

[B23] Sanseverino W Roma G De Simone M Faino L Melito S Stupka E Frusciante L Ercolano MR PRGdb: a bioinformatics platform for plant resistance gene analysis Nucleic Acids Res 2010 38 Database issue D814 821 19906694 10.1093/nar/gkp978 PMC2808903

[B24] Martin GB Bogdanove AJ Sessa G Understanding the functions of plant disease resistance proteins Annu Rev Plant Biol 2003 54 23 61 10.1146/annurev.arplant.54.031902.135035 14502984

[B25] Yang S Bourne PE The evolutionary history of protein domains viewed by species phylogeny PLoS One 2009 4 12 e8378 10.1371/journal.pone.0008378 20041107 PMC2794708

[B26] Zhang Q Zmasek CM Dishaw LJ Mueller MG Ye Y Litman GW Godzik A Novel genes dramatically alter regulatory network topology in amphioxus Genome Biol 2008 9 R123 10.1186/gb-2008-9-8-r123 18680598 PMC2575513

[B27] Yue JX Meyers BC Chen JQ Tian D Yang S Tracing the origin and evolutionary history of plant nucleotide-binding site-leucine-rich repeat (NBS-LRR) genes New Phytol 2012 193 4 1049 1063 10.1111/j.1469-8137.2011.04006.x 22212278

[B28] Jones DA Takemoto D Plant innate immunity - direct and indirect recognition of general and specific pathogen-associated molecules Curr Opin Immunol 2004 16 1 48 62 10.1016/j.coi.2003.11.016 14734110

[B29] Moffett P Farnham G Peart J Baulcombe D Interaction between domains of a plant NBS-LRR protein in disease resistance-related cell death EMBO J 2002 21 17 4511 10.1093/emboj/cdf453 12198153 PMC126192

[B30] Zhu M Shao F Innes RW Dixon JE Xu Z The crystal structure of Pseudomonas avirulence protein AvrPphB: a papain-like fold with a distinct substrate-binding site Proc Natl Acad Sci USA 2004 101 1 302 307 10.1073/pnas.2036536100 14694194 PMC314180

[B31] Rooney HC Van't Klooster JW Van der Hoorn RA Joosten MH Jones JD De Wit PJ Cladosporium Avr2 inhibits tomato Rcr3 protease required for Cf-2-dependent disease resistance Science 2005 308 5729 1783 1786 10.1126/science.1111404 15845874

[B32] Stone JM Walker JC Plant protein kinase families and signal transduction Plant Physiol 1995 108 2 451 457 10.1104/pp.108.2.451 7610156 PMC157363

[B33] Tameling WI Elzinga SD Darmin PS Vossen JH Takken FL Haring MA Cornelissen BJ The tomato R gene products I-2 and MI-1 are functional ATP binding proteins with ATPase activity Plant Cell 2002 14 11 2929 2939 10.1105/tpc.005793 12417711 PMC152737

[B34] Kopp EB Medzhitov R The Toll-receptor family and control of innate immunity Curr Opin Immunol 1999 11 1 13 18 10.1016/S0952-7915(99)80003-X 10047546

[B35] Collier SM Moffett P NB-LRRs work a "bait and switch" on pathogens Trends Plant Sci 2009 14 10 521 529 10.1016/j.tplants.2009.08.001 19720556

[B36] Dievart A Clark SE LRR-containing receptors regulating plant development and defense Development 2004 131 2 251 261 14701679 10.1242/dev.00998

[B37] Panstruga R Discovery of novel conserved peptide domains by ortholog comparison within plant multi-protein families Plant Mol Biol 2005 59 3 485 500 10.1007/s11103-005-0353-0 16235112

[B38] Kobe B Deisenhofer J Crystal structure of porcine ribonuclease inhibitor, a protein with leucine-rich repeats Nature 1993 366 6457 751 756 10.1038/366751a0 8264799

[B39] Finn RD Mistry J Schuster-Bockler B Griffiths-Jones S Hollich V Lassmann T Moxon S Marshall M Khanna A Durbin R Pfam: clans, web tools and services Nucleic Acids Res 2006 34 Database issue D247 251 16381856 10.1093/nar/gkj149 PMC1347511

[B40] McHale L Tan X Koehl P Michelmore RW Plant NBS-LRR proteins: adaptable guards Genome Biol 2006 7 4 212 10.1186/gb-2006-7-4-212 16677430 PMC1557992

[B41] Deslandes L Olivier J Theulieres F Hirsch J Feng DX Bittner-Eddy P Beynon J Marco Y Resistance to Ralstonia solanacearum in Arabidopsis thaliana is conferred by the recessive RRS1-R gene, a member of a novel family of resistance genes Proc Natl Acad Sci USA 2002 99 4 2404 2409 10.1073/pnas.032485099 11842188 PMC122377

[B42] Xu Z Ramakrishna W Retrotransposon insertion polymorphisms in six rice genes and their evolutionary history Gene 2008 412 1–2 50 58 18291601 10.1016/j.gene.2008.01.012

[B43] Stange C Matus JT Dominguez C Perez-Acle T Arce-Johnson P The N-homologue LRR domain adopts a folding which explains the TMV-Cg-induced HR-like response in sensitive tobacco plants J Mol Graph Model 2008 26 5 850 860 10.1016/j.jmgm.2007.05.006 17631403

[B44] Hunter S Apweiler R Attwood T Bairoch A Bateman A Binns D Bork P Das U Daugherty L Duquenne L InterPro: the integrative protein signature database Nucleic Acids Res 2009 37 Database issue D211 215 18940856 10.1093/nar/gkn785 PMC2686546

[B45] Ihaka RG R R: A language for data analysis and graphics J Comput Graph Stat 1996 5 15

[B46] Kall L Krogh A Sonnhammer E Advantages of combined transmembrane topology and signal peptide prediction--the Phobius web server Nucleic Acids Res 2007 35 Web Server issue W429 432 17483518 10.1093/nar/gkm256 PMC1933244

[B47] Krogh A Larsson B von Heijne G Sonnhammer EL Predicting transmembrane protein topology with a hidden Markov model: application to complete genomes J Mol Biol 2001 305 3 567 580 10.1006/jmbi.2000.4315 11152613

[B48] Edgar RC MUSCLE: multiple sequence alignment with high accuracy and high throughput Nucleic Acids Res 2004 32 5 1792 1797 10.1093/nar/gkh340 15034147 PMC390337

[B49] Anisimova M Gascuel O Approximate likelihood-ratio test for branches: A fast, accurate, and powerful alternative Syst Biol 2006 55 4 539 552 10.1080/10635150600755453 16785212

[B50] Bai Y Pavan S Zheng Z Zappel NF Reinstadler A Lotti C De Giovanni C Ricciardi L Lindhout P Visser R Naturally occurring broad-spectrum powdery mildew resistance in a Central American tomato accession is caused by loss of mlo function Mol Plant Microbe Interact 2008 21 1 30 39 10.1094/MPMI-21-1-0030 18052880

